# A mutational analysis and molecular dynamics simulation of quinolone resistance proteins QnrA1 and QnrC from *Proteus mirabilis*

**DOI:** 10.1186/1472-6807-10-33

**Published:** 2010-10-08

**Authors:** Qinglan Guo, Jingwei Weng, Xiaogang Xu, Minghua Wang, Xiaoying Wang, Xinyu Ye, Wenning Wang, Minggui Wang

**Affiliations:** 1 Institute of Antibiotics, Huashan Hospital Fudan University, 12 Wulumuqi Road, Shanghai 200040, China; 2 Department of Chemistry, Fudan University,220 Handan Road, Shanghai 200433, China; 3 Institute of Biomedical Sciences, Shanghai Key Laboratory of Molecular Catalysis and Innovative Materials, Fudan University, 138 Yixueyuan Road, Shanghai 200032, China

## Abstract

**Background:**

The first report on the transferable, plasmid-mediated quinolone-resistance determinant *qnrA1 *was in 1998. Since then, *qnr *alleles have been discovered worldwide in clinical strains of Gram-negative bacilli. Qnr proteins confer quinolone resistance, and belong to the pentapeptide repeat protein (PRP) family. Several PRP crystal structures have been solved, but little is known about the functional significance of their structural arrangement.

**Results:**

We conducted random and site-directed mutagenesis on *qnrA1 *and on *qnrC*, a newly identified quinolone-resistance gene from *Proteus mirabilis*. Many of the Qnr mutants lost their quinolone resistance function. The highly conserved hydrophobic Leu or Phe residues at the center of the pentapeptide repeats are known as *i *sites, and loss-of-function mutations included replacement of the *i *site hydrophobic residues with charged residues, replacing the *i*^-2 ^site, N-terminal to the *i *residues, with bulky side-chain residues, introducing Pro into the β-helix coil, deletion of the N- and C-termini, and excision of a central coil. Molecular dynamics simulations and homology modeling demonstrated that QnrC overall adopts a stable β-helix fold and shares more similarities with MfpA than with other PRP structures. Based on homology modeling and molecular dynamics simulation, the dysfunctional point mutations introduced structural deformations into the quadrilateral β-helix structure of PRPs. Of the pentapeptides of QnrC, two-thirds adopted a type II β-turn, while the rest adopted type IV turns. A gap exists between coil 2 and coil 3 in the QnrC model structure, introducing a structural flexibility that is similar to that seen in MfpA.

**Conclusion:**

The hydrophobic core and the β-helix backbone conformation are important for maintaining the quinolone resistance property of Qnr proteins. QnrC may share structural similarity with MfpA.

## Background

Quinolones constitute an important group of antimicrobials active against Gram-negative and Gram-positive bacteria. Because of wide clinical use, clinical isolates resistant to fluoroquinolone are emerging and spreading rapidly. In China, more than 60% of *Escherichia coli *strains isolated from hospital-acquired infections are resistant to fluoroquinolone, and 50.6% of *E. coli *strains from community-acquired infections are ciprofloxacin-resistant [1]. The resistance mechanism of these drugs was considered to be chromosomally encoded until the discovery of the plasmid-mediated *qnrA *gene in 1998 [2]. Thereafter, additional *qnr *genes (*qnrA*, *qnrB*, *qnrS*, *qnrD*) on resistance plasmids were identified worldwide, in various bacterial pathogens. The chromosomes of *Vibrionaceae*, *Stenotrophomonas maltophilia*, and Gram-positive genera were found to contain *qnr*-like genes [3-5]. More recently, our research group reported a new *qnr *gene, *qnrC*, found in a clinical strain of *Proteus mirabilis *[6].

**Figure 1:**
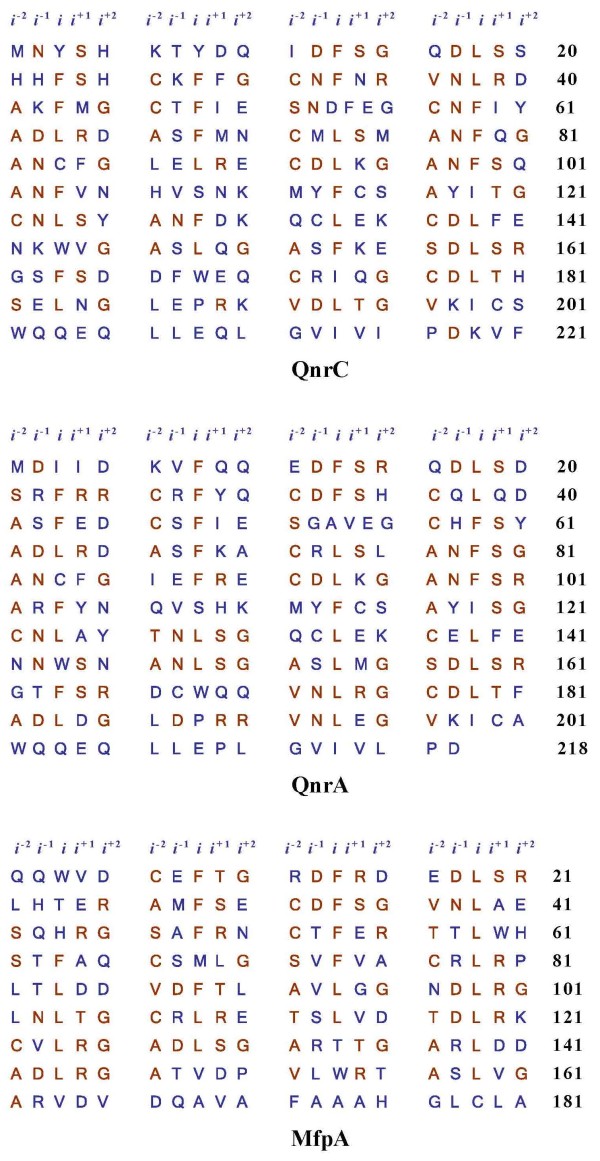
**The pentapeptide repeats of QnrC, QnrA1 and MfpA**. The characteristic pentapeptide repetitions are [A/C/S/T/V] [D/N] [L/F] [S/T/R] [G/R], highlighted in red. The conserved hydrophobic residue at the center of pentapeptide alignments is designated as *i*, while the residues N-terminal to the *i *residue are designated as *i*^-1 ^and *i*^-2 ^and the residues C-terminal to the *i *residue as *i*^+1 ^and *i*^+2^.

The Qnr proteins belong to the pentapeptide repeat protein (PRP) family. The QnrA protein competes with DNA for binding to DNA gyrase [7,8], suggesting that QnrA may provide quinolone resistance by acting as a DNA mimic. PRP proteins, which contain characteristic tandem pentapeptide repeats [A/C/S/T/V] [D/N] [L/F] [S/T/R] [G/R] [[3,7,9,10], Fig. 1], are most abundant in cyanobacteria, and are widely distributed in prokaryotes [9]. The highly conserved hydrophobic residues (Leu or Phe) at the center of the pentapeptide repeats are usually designated as site *i*, with the residues N-terminal to *i *as the *i*^-2 ^and *i*^-1 ^sites, and the residues C-terminal to *i *as the *i*^+1 ^and *i*^+2 ^sites [Fig. 1]. To date, only six PRP family crystal structures have been determined [11-16]. These structures showed that all PRPs adopt a right-handed quadrilateral β-helix (RHQBH) fold. Every four pentapeptide repeats form a nearly square repeating unit, termed a coil. The coils are stacked atop one another to facilitate hydrogen bonding between neighboring coils. The two predominant main chain conformations encoded by the pentapeptide repeat sequence differ only in the orientation of a single peptide bond between residue *i *and *i*^+1^. In the type II turns composed of the *i*, *i*^+1^, *i*^+2 ^and *i*^-2 ^residues, the main chain *ϕ*-*ψ *angles of residues in *i *and *i*^+1 ^are (-120, 20) and (-60, 120) respectively, while in the type IV turns the *ϕ*-*ψ *angles of residue *i *and *i*^+1 ^are (-120, 120) and (-120, 120). The residue side chains are also regularly positioned. The residues at site *i *and *i*^-2 ^are packed inside the β-helix forming a hydrophobic core, while the residues at sites *i^-1^*, *i^+1 ^*and *i^+2 ^*are exposed to solvent [9]. Among the resolved structures of PRPs, MfpA [[11], 2bm4] and *Efs*Qnr [[15], 2w7z] present a unique rod-shaped dimer form, in which the two monomers associate through their C-terminal helices. This dimer assembly was proposed to be a DNA mimic, and shown to be capable of binding to DNA gyrase *in vitro *[11]. MfpA is a good model for Qnr proteins, as they all possess a characteristic PRP sequence and share the same target protein [7,11,17]. Although the sequence and structural characteristic of PRPs have been determined, the relationship between their structure and function remains elusive.

In this study, mutagenesis was carried out on different parts of QnrC and QnrA1, to explore the functional importance of the targeted residues. Many mutants were found to lose their ability to confer ciprofloxacin resistance. Combined with computational simulations and homology modeling, we found that the hydrophobic core and the β-helix backbone conformation are important for the quinolone resistance function of QnrC and QnrA1.

## Results

### 1. Quinolone resistance phenotypes of QnrC and QnrA1 mutants

*E. coli *TOP10 cells harboring wild type *qnrC *in plasmid pHS12, or wild type *qnrA1 *in plasmid pMG252-1 both had a minimal inhibitory concentration (MIC) for ciprofloxacin of 0.125 μg ml^-1 ^(Table 1). Mutants of these genes were classified into four groups.

**Table 1:** Ciprofloxacin susceptibility of QnrC and QnrA1 mutants.

Groups/clusters	Mutagenesis and codon alteration ^a^	Position of mutation	Qnr protein	MIC (μg ml^-1^)
	pHSG398	-	-	0.002
	
Control	pHS12	wild type	QnrC	0.125
	
	pMG252-1	wild type	QnrA1	0.125
	

	L38R(TTA→AGA)	i	QnrC	0.003
	
	L38A(TTA→GCA)	i	QnrC	0.125
	
Hydrophobic interior of β-helix	L38F(CTG→TTC)	i	QnrA1	0.125
	
	F13S(TTC→TCC)^c^	i	QnrA1	0.008
	
	C72Y(TGC→TAC) or A97Y(GCT→TAT)	i^-2^	QnrC	0.003

	S116P (TCT→CCT) ^c^	i^+2^	QnrC	0.002
	
Constraint effect of Pro to the backbone	S153P(TCT→CCT)^c^	i^-1^	QnrC	0.003
	
	L38P(CTG→CCG)^c^	i	QnrA1	0.004

	C84S(TGT→TCT)	i	QnrC	0.094
	
	C84S(TGC→AGC)	i	QnrA1	0.064
	
Cys to Ser mutation	C31 S or C36 S or C57 S or C177 S (TGT→AGT); C26 S or C46 S or C72 S or C92 S or C122 S or C137S(TGC→AGC)	i^-2^	QnrA1	0.125
	
	C133S(TGC→AGC)or C168S(TGT→AGT)	i^-1^	QnrA1	0.125
	
	C115S(TGC→AGC)or C200S(TGT→AGT)	i^+1^	QnrA1	0.125

	Δ11-20	N-terminus	QnrC	0.003
	
	Δ2-21	N-terminus	QnrA1	0.002
	
	Δ2-10	N-terminus	QnrA1	0.003
	
	Δ49-55	G56 region	QnrC	0.003
	
Fragment truncation b	Δ41-56, Δ51-56	G56 region	QnrA1	0.003
	
	Δ77-96, Δ137-156	β-helix backbone	QnrC	0.003
	
	Δ216-218	C-terminus	QnrC	0.064
	
	Δ187-218	C-terminus	QnrA1	0.003
	
	Δ207-218	C-terminus	QnrA1	0.004

	D188V (GAC→GTC)^c^	i^+1^	QnrA1	0.003
	
Others	M44T(ATG→ACG)	i^+1^	QnrC	0.125
	
	I216T (ATT→ACT) ^c^	i^+2^	QnrC	0.125
	
	E50A(GAA→GCC) or E50G (GAA→GGC) or E55A (GAA→GCT) or E55G(GAA→GGA)	i^+2^	QnrA1	0.125

^a ^nucleotide substitution is in parentheses

^b ^Δ2-21 indicates deletion of amino acids 2-21

^c ^mutations were obtained by random mutagenesis and confirmed by site-directed mutagenesis

#### 1) Mutations in *i *or *i*^-2^

In all known PRP crystal structures, the residues at *i *and *i*^-2 ^have side chains that pack inward, forming a hydrophobic core of RHQBH. When the hydrophobic residues at *i *were substituted with residues with polar or charged side chains, the ciprofloxacin MICs decreased. For example, mutation F13 S had an MIC of 0.003 μg ml^-1^, and L38R had an MIC of 0.008 μg ml^-1^, indicating loss of quinolone resistance. The control, which was substitution of *i *with neutral or hydrophobic side chain residues such as L38A or L38F, did not reduce MICs (Table 1). At *i*^*-*2^, substitution of Tyr, which has a bulky side chain, for Cys or Ala, as in the C72Y and A97Y mutants, completely destroyed activity. These data indicated that the *i *region excludes polarized and charged residues, and *i*^*-2 *^tends to be sensitive to the presence of bulky side chains.

#### 2) Introduction of proline

All random mutants lost activity when Pro was introduced into the pentapeptide repeat sequence in the *i*, *i*^*-1 *^or *i*^+2 ^regions, as seen in mutations L38P, S116P, and S153P (Table 1).

#### 3) Cys to Ser mutations

Cys residues are abundant in Qnr proteins, relative to other PRPs. We introduced single point mutations of Cys to Ser to perform a complete search of potential disulfide bonds in PRPs [13]. The mutations all involved Cys in QnrA1, in *i*^*-2 *^(residues 26, 31, 36, 46, 57, 72, 92, 122, 137), *i*^*-1 *^(residue 133), *i *(residue 84), *i*^*+1 *^(residue 115), and in some non-pentapeptide repeat residues (residues 168, 177, 200). Most of these mutations showed little variation in MIC values, regardless of whether the mutated side chains were originally inward-facing (position *i*^*-2*^) or outward-facing (position *i*^*-1 *^and *i*^*+1*^). Only the C84 S mutants showed an obvious decrease in ciprofloxacin MIC, from 0.125 μg ml^-1 ^to 0.064 for QnrA1. When we introduced a C84 S mutation into QnrC, the MIC decreased to 0.094 μg ml^-1 ^(Table 1). Therefore, replacement of the sulfhydryl group with a hydroxyl group was tolerated, suggesting that either no disulfide bond was formed at the site, or a disulfide bond was formed, but was not essential for activity.

#### 4) Fragment truncation of Qnr proteins

The N-, and C-terminal residues, and the coils in the middle of the β-helix were truncated to determine their potential functional importance. Ciprofloxacin MIC values showed that increased susceptibility resulted from removal of residues 2-21, 2-10, and 11-20 at or near the N-terminus of the Qnr proteins; residues 187-218, 207-218 and 216-218 at the C-terminus; residues 41-56, 49-55, and 51-56 around the G56 region; or removal of residues 77-96 and 137-156, corresponding to the intact coils of the β-helix (Table 1).

#### 5) Other mutations

The D188V mutation proximal to the C-terminus of QnrA1 conferred increased susceptibility to ciprofloxacin. The mutations M44T, I216T, E50G, E50A, E55G and E55A did not affect quinolone resistance activity (Table 1).

### 2. Molecular dynamics simulations

#### 1) Stability of wild-type MfpA structure

A 10-ns molecular dynamics (MD) trajectory revealed that the structure of the wild-type MfpA dimer has high stability. The typical right-handed quadrilateral β-helix (RHQBH) backbone of each monomer varied little, with the C_α _root-mean-square deviation (RMSD) value fluctuating around 0.8 Å throughout the simulation. The hydrophobic core inside the β-helix remained stable, and the hydrogen networks between coils were also well preserved. In spite of the stable conformation, the individual monomers underwent obvious relative bending motions around the hinge at the dimer interface. However, this motion did not disrupt the connections between the monomers, which included the hydrogen bond network between G161, A162, R163 and V164 at the last β-helix coil of one monomer, and G177 and C179 at the C-terminus of the opposite monomer. The van der Waals interactions between the hydrophobic side chains of the C-terminal α-helices also contributed to the connection. Overall, the C-terminus of each monomer seemed to be essential for MfpA dimer assembly.

#### 2) Mutations at site *i*

**Figure 2:**
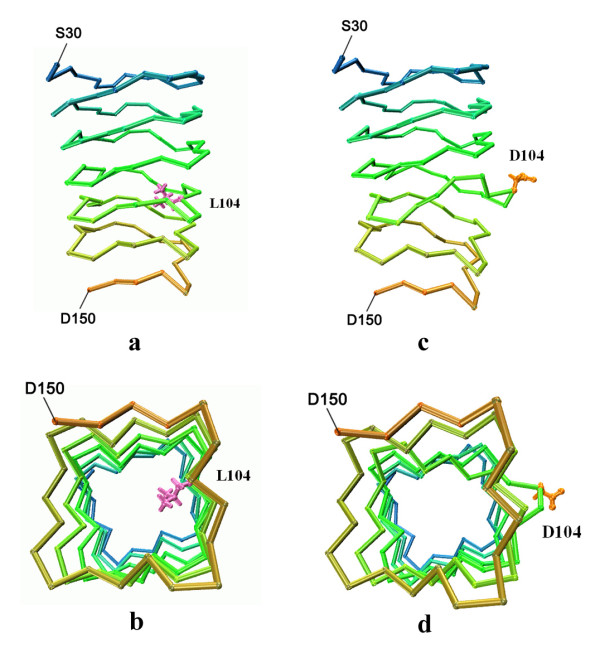
**Structure perturbation induced by L104 D mutation in MfpA**. **(a) **Part of the structure of wild type MfpA at the end of 10-ns MD simulation. The side chain of L104 (magenta) is packed in the hydrophobic core of the β helix; **(b) **bottom view of **(a)**; **(c) **Part of the structure of MfpA L104 D mutant at the end of 10-ns MD simulation. The side chain of D104 (orange) is exposed to solvent, inducing local structure deformation; **(d) **bottom view of **(c)**.

The high conservation at the *i *site implied its importance to the structure and function of PRPs. The Phe or Leu residues form a hydrophobic core within the protein, so we examined the structural variations of two Leu to Asp mutants to see the effect of a strong polar side chain at the *i site*. In both L39 D and L104 D mutants, the acidic side chains showed a strong tendency to escape from the hydrophobic core, and the nearby backbones also distorted distinctly from the typical RHQBH structure. The L104 D mutant exhibited more striking variation. The D104 side chain flipped over from the initial orientation pointing toward the hydrophobic core to face the solvent environment (Fig. 2d). This reorientation occurred just after the beginning of the simulation. In contrast, the wild-type L104 alkyl chain remained oriented toward the interior space throughout the 10-ns trajectory (Fig. 2b). The reorientation of the side chains distorted the local backbone in the vicinity of D104 (Fig. 2d). This enlarged the coil-coil distance, creating a gap between them. The local hydrogen bond networks between the coils were also disrupted. These structural changes indicated that the hydrophobic core could not accommodate charged residues at the *i *site, verifying the importance of the conserved hydrophobic residues to PRP structural stability. This is consistent with the observed dysfunction of the F13 S and L38R mutants.

#### 3) Constraining effect of proline on the backbone

**Figure 3:**
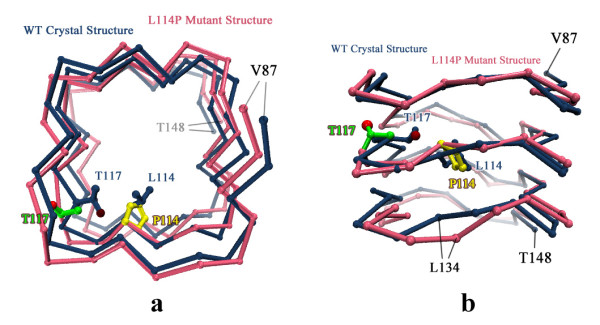
**Structure perturbation induced by L114P mutation in MfpA**. Top view (a) and side view (b) of the superimposed wild type (blue) and L114P mutant (pink) structures after 10-ns MD simulation. Only part of the structure (residue 87 to 148) is shown. The side chain of T117 in the L114P mutant is in green, with red balls for hydroxyl oxygen atom. The side chain of P114 residue is in yellow.

Pro mutations caused dysfunction, as observed with the L38P, S116P and S153P mutants. This might be attributed to the conformational restraints of proline residues on the protein backbone. We introduced Pro at L114 (position *i*) in MfpA and found that the protein was substantially perturbed in two ways. First, we noted an increased distance between P114 and T134, a residue within *i *of the neighboring coil (Fig. 3). This could be largely attributed to the missing backbone amide in P114, leading to the disruption of the original backbone hydrogen bond between the L114 amide and the K133 carbonyl in the wild type protein. Thus, the intercoil interaction was weakened and the fluctuation amplitude of coils increased. The second remarkable change was around T117 (position *i*^*-*2^). In the wild type protein, the T117 hydroxyl group is buried in the β-turn region to form hydrogen bonds with the backbone of L104 and V105. The L114P mutation changed the T117 side chain orientation. The hydroxyl group of T117 pointed towards N97, and interacted with its carbonyl. To accommodate the side chain rearrangements, the backbone distorted, resulting in the increased distance between T117 and its preceding coil (Fig. 3).

### 3. Homology modeling of QnrC protein

**Figure 4:**
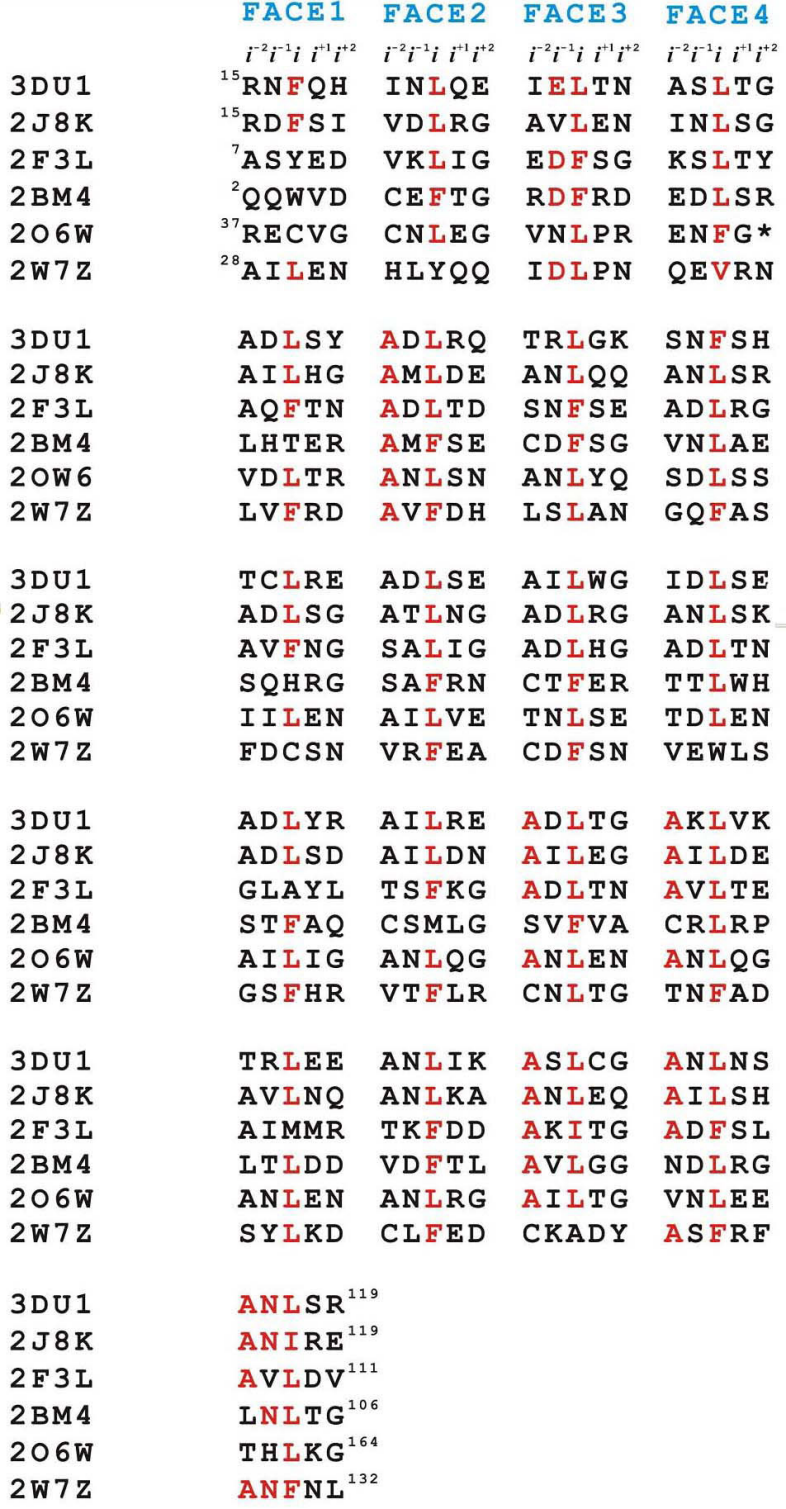
**Structure-based sequence alignment of the six PRP proteins with known structures showing the sequence diversity**. Conserved residues are highlighted in red.

Several crystal structures of PRPs have been reported, but no structure template had a sequence identity with QnrC higher than 30%. However, structural studies so far have revealed that the pentapeptide repeat sequences in PRPs adopt a right-handed quadrilateral β-helix fold, despite the sequence diversity (Fig. 4). Based on these observations, we inferred that the pentapeptide repeat sequences in QnrC fold into a similar structure. However, the pentapeptide repeats make conventional sequence alignment difficult. Nonetheless, the periodic structural feature of the quadrilateral β-helix ensures that the general location and conformation of the side chains and intercoil interactions are conserved among different alignments. If we confine the model building within the regular pentapeptide repeat sequence, the model structure may have higher reliability than expected from the sequence similarity between the target and template. We excluded the C-terminal part of QnrC (166-221) from model building because of the lack of a regular pentapeptide repeat. As a template, we used the crystal structure of the pentapeptide repeat protein Np275/276 (PDBID: 2J8K), from which the N- and C-terminal regions (1-14, 168-175), which deviate from the regular quadrilateral β-helix structure, were removed. After truncation, the template was 11 amino acids shorter than QnrC. Therefore, a fragment of the previous coil (from H148 to T158) was duplicated and added after L167. Within QnrC (1-165) however, the regular pentapeptide repeat is disrupted by an abnormal six-residue motif (^51^SNDFEG^56^), which brings uncertainty into the model building. Therefore, we simply aligned the six-residue motif in one quadrilateral face in the initial homology model building, and subjected this to optimization using MD simulation.

**Figure 5:**
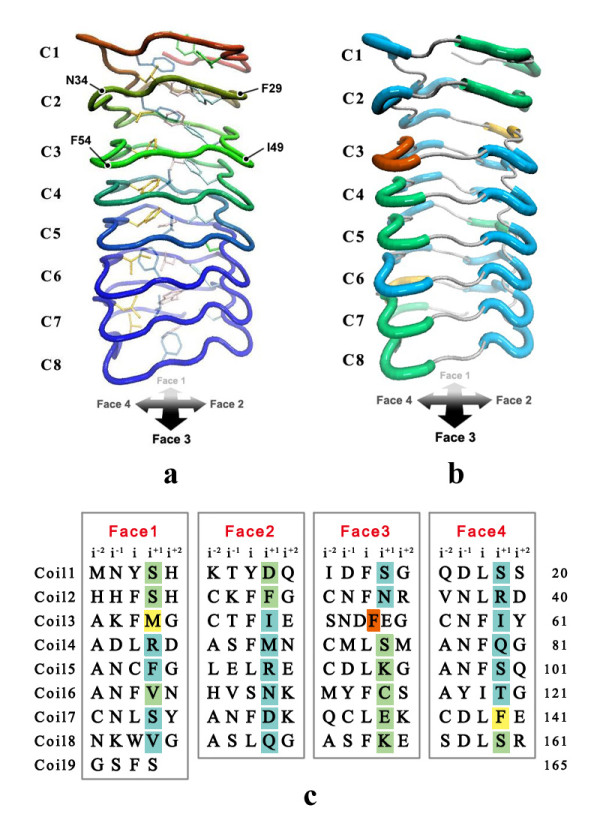
**The structure of QnrC protein (residue 1 to 165) based on homology modeling**. (a) Tube representation with all side chains of residues at position *i*. The structure adopts a β-helix fold. Leu and Phe at *i *positions are well aligned at Face 1, 2, 3 and 4. (b) and (c) illustrate the distribution of type II (in blue) and type IV (in green) β-turns formed by residues at position *i^+1^*, *i^+2 ^*and the residue at position *i^-2 ^*in the subsequent pentapeptide in the model structure (b) and in sequence (c). The turn involved in the hexapeptide segment (F54 to C57) is in orange. The two turns (M44 to C46 and F140 to N142) that switch between type II and type IV frequently during the structure optimization MD simulation are highlighted in yellow.

The homology model structure of QnrC (residue 1 to 165) optimized by 10-ns MD simulation retained the orderly β-helix fold overall (Fig. 5a). The highly conserved Phe and Leu residues in the *i *site retained van der Waals contact with each other and with the neighboring coils, that formed the hydrophobic core. The coils stacked atop one another and were stabilized by hydrogen bond interactions. Nearly two-thirds of pentapeptides adopted type II β-turns with the carbonyl of an *i *residue hydrogen bonding to the amide of an *i^-2 ^*residue in the following pentapeptide. The rest of the pentapeptides adopted type IV β-turns, in which the main chain atoms of both *i *and *i*^+1 ^residues participated in intercoil hydrogen bonding. Some of the type IV turns were located near the N-terminus (Fig. 5b and 5c), as observed in MfpA [11]. We also noted that most turns on face 3 were type IV, while the other faces were dominated by type II turns. The distribution of the two types of β-turns is proposed to be related to the sequence identity of the residues at specific positions [9].

**Figure 6:**
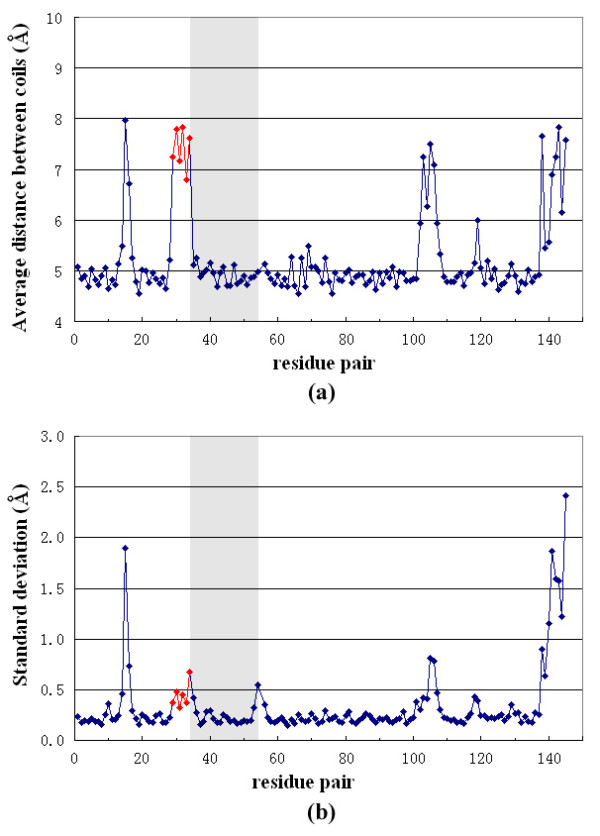
**Intercoil distance variations of QnrC along the MD simulation trajectory**. The intercoil distance is defined as the distance between Cα atoms of residue (*i*) and residue (*i*+20) in the subsequent coil. (Note that for residues N34 to F54 [shadowed], the distance is between Cα [*i*] to Cα [*i*+21].) The average value **(a) **and the standard deviation **(b) **of the distances were calculated for the last 10 ns of the MD simulation. The points involved in the gap between coil 2 and coil 3 are in red.

After optimization by MD simulation, the abnormal six-residue motif (51 to 56) was seen to introduce structural deviations from the typical β-helix arrangement, including a large separation between coil 3 and coil 2. The hydrogen bonds between the backbone of the hexapeptide and its following coil were well preserved, while the initial hydrogen bonds between the hexapeptide and coil 2 disappeared because of the large intercoil distance. (Fig. 6a). Along the MD simulation trajectory of QnrC, the intercoil distance between coil 3 and coil 2 near the gap underwent much larger fluctuations than the rest of the protein (Fig. 6b), implying additional structural flexibility in QnrC.

The C-terminal part of QnrC was not included in our homology modeling because of the lack of a template with high sequence homology. However, secondary structure prediction showed an additional α-helix near the very C-terminus (200~210 aa), implying a similar structural arrangement of QnrC with MfpA at this region. Taken together, the partial model structure of QnrC based on homology modeling and MD simulation suggested an overall structural arrangement and features of QnrC that may be highly similar to MfpA. However, we note uncertainties in this model structure, and analysis awaits verification from future structural studies.

## Discussion

The *qnr *gene and its variants carried by plasmids are widely distributed in clinical isolates, and provide low-level quinolone resistance. To date, more than 30 *qnr *alleles have been identified, with seven QnrA alleles, four QnrS alleles, twenty-four QnrB alleles, one QnrC and one QnrD, and more than 50 amino acid alterations described [[18], http://www.lahey.org/qnrStudies]. However, little is known about the potential influence of single mutations on Qnr protein function. Random mutagenesis was carried out by Cattoir et al., who found no mutants of QnrA or QnrS with an elevated MIC for quinolones. In contrast, MICs for quinolones for C115Y mutations decreased 2.5-to-5-fold relative to wild type strains [19]. Rodríguez-Martínez et al. found only one mutant with increased quinolone resistance: QnrS1, which contains D185Y with four folds for ciprofloxacin [20]. Mutations at G56-, G56 D, C72Y, C92Y, G96 D, or L159 D in QnrA1, QnrB1, or QnrS1 cause reduced activity for ciprofloxacin [20]. In this study, no random mutagenesis QnrC or QnrA1 mutants were found to have elevated resistance to ciprofloxacin. Synonymous mutations occurred frequently, along with some non-synonymous mutants with unchanged function. This is consistent with the high diversity of Qnr variants in clinical isolates. Many mutants have disabled ciprofloxacin resistance activity caused by only a single amino acid substitution for a conserved or unconserved residue. Examples include substitution of the conserved hydrophobic Leu or Phe residues with polar or positively charged residues (L38R, F13S) in the *i *site, residues with small side chains substituted with residues with bulky side chains (C72Y, A97Y) in the *i*^-2 ^site, and charged residues substituted for non-polar residues (D188V). In addition, when a Pro occurred in the regular β-helix at the *i, i*^-1^, or *i*^+2 ^sites, the Qnr mutants tended to be susceptible to ciprofloxacin.

To understand the functional consequence of our mutagenesis experiments, we performed *in silico *mutations based on the MfpA structure, and subjected them to MD simulation. The MD simulation of the wild type MfpA dimer demonstrated that the dimer assembly has large intermonomer motion in solution. This implied that the protein has an advantageous structural flexibility for target recognition or interaction. The L39 D and L104 D mutations of MfpA perturb the hydrophobic residues at the *i *site. As expected, charged residues at the *i *site were not tolerated by the hydrophobic core of the RHQBH. The reorientation of the charged side chain to the solvent accessible side induced an intercoil gap in the vicinity of the mutated residues. Similarly, the L114P MD simulation indicated that the introduction of a Pro residue to the β-helix structure increased the intercoil gap in the proximity of the mutated residue. Some of the intercoil hydrogen bonds were broken and the structural flexibility around the mutated position increased remarkably (Fig. 6). Homology modeling of QnrC gave a similar intercoil disruption at the hexapeptide sequence, which showed significant structural flexibility. These structural perturbations to the RHQBH are very similar to the intercoil disruption caused by cis-P81 in wild type MfpA. Although the functional implication of intercoil disruption near P81 is not clear, it may be a critical structural feature for the Qnr and MfpA protein family. We note however, that MD simulation at tens of ns may not be able to efficiently sample all possible larger structural changes in both the mutation systems and homology model structure. Another limitation of the simulation is that point mutations are based on the MfpA structure. Therefore, additional biochemical and structural characterizations are needed to address this issue.

The structure of the MfpA dimer exhibits characteristics similar to B-form DNA in size, shape, and electronegative surface potential, and fits comfortably in the DNA-binding surface of DNA gyrase [11,21], suggesting a DNA mimicking mechanism of drug resistance. Many Qnr mutants are defective in fluoroquinolone resistance, so we propose that the resistance mechanism for Qnr and MfpA is different from enzymes that have an active site or catalytic domain. Qnr proteins protect bacteria from fluroquinolone attack by inhibiting the activity of DNA gyrase, thus slowing down the growth of bacteria [22]. Therefore enhanced function of Qnr proteins is likely to be lethal to the host cell. This may explain why few Qnr mutants have been found that have elevated MIC values. Quinolone resistance of the Qnr protein likely developed as a secondary function of PRP gene products, and the physiological role of PRPs in prokaryotic cells remains elusive [11].

## Conclusions

In this study, several Qnr mutants with defective activity were obtained by random or site-directed mutagenesis, but none had enhanced function. The conserved PRP residues at the *i *and *i*^-2 ^sites were of great importance to Qnr protein function. The introduction of Pro to the β-helix caused protein dysfunction. The C- and N-termini, and the G56 region were also crucial to Qnr protein function. Molecular dynamics simulations and homology modeling revealed that QnrC adopts a stable β-helix fold with strong structural similarity to MfpA. Both QnrC and MfpA showed significant structural flexibility that might be favorable to target recognition or interaction.

## Methods

### Construction of random mutation libraries of qnrC and qnrA1

The *qnrC*-carrying plasmid pHS10 was isolated from a clinical strain of *P. mirabilis *06-489 from Huashan Hospital, a teaching hospital at Fudan University in Shanghai [6]. The *qnrA1 *carried by plasmid pMG252 was donated by Professor G.A. Jacoby [2]. Random mutations were generated in the *qnrC *and *qnrA1 *genes using the Genemorph II Random Mutagenesis Kit (Stratagene, La Jolla, CA, USA.). Error-prone 904 bp and 921 bp PCR fragments encompassing the entire transcription units of *qnrC *and *qnrA1 *were amplified with pHS10 or pMG252 as the templates, and primers qnrCBam/qnrCSal [6] and qnrA1Sal/qnrA1Eco (Table 1). PCR products were cloned into pHSG398, containing Chl^r ^conferring resistance to chloramphenicol (Takara Bio, Otsu, Japan), and recombinants transformed into *E. coli *TOP10 (Invitrogen) with selection on tryptic soy agar (TSA, Oxoid, Basingstoke, England) plates containing chloramphenicol 34 μg ml^-1^. The resultant plasmids were isolated, and inserts were verified by sequencing. Wild type recombinants containing *qnrC *and *qnrA1 *genes were called pHS12 [6] and pMG252-1.

### Site-directed mutagenesis of qnrC and qnrA1

To explore the potential function of the N- and C-termini, and the G56 region of Qnrs, nucleotide deletions were introduced into *qnrC *and *qnrA1 *for multiple amino acid deletions using a QuikChange site-directed mutagenesis kit (Stratagene, La Jolla, CA). Complementary primers with the desired mutation were designed, flanked by unmodified nucleotide sequence using Stratagene's web-based QuikChange Primer Design Program (http://www.stratagene.com/qcprimerdesign, see Additional file 1). Mutagenesis used the experimental protocol of the manufacturer. For example, primers used for deletion mutation of residue 11-20 for QnrC were QnrC-Δ11-20-F (5'- CCCATAAAACGTACGATCAA---CATCACTTTTCTCACTG -3') and QnrC-Δ11-20-R (5'- CAGTGAGAAAAGTGATG---TTGATCGTACGTTTTATGGG -3'). Dashes indicate deleted nucleotides that encode amino acids 11-20 of QnrC. For whole-plasmid amplificaiotn, 5-50 ng of recombinant plasmid pHS12 or pMG252-1 was used as template in a QuikChange amplification reaction with PfuTurbo DNA polymerase. PCR products were digested with Dpn I restriction enzyme at 37°C for 1 hour, then 1 μ-l of the Dpn I-treated DNA was transferred to E. coli TOP10 competent cells. Transformants were selected with chloramphenicol, and mutations were verified by DNA sequencing. Single amino acid substitutions at the *i *and *i*^-2 ^sites and in other conserved residues of Qnr, and codons for mutant residues are shown in Table 1.

### Susceptibility testing

MICs of ciprofloxacin for random or site-directed mutants were determined by CLSI agar dilution methodology [23]. Mutants with elevated or decreased MICs were confirmed by E test (Biodisk AB, Solna, Sweden).

### Molecular dynamics simulation

Software package NAMD 2.6 [24] was employed for MD simulation using CHARMM27 force field. Simulation conditions were maintained at 1.01325 bar by the Nòse-Hoover Langevin piston method [25] and 300 K by Langevin dynamics [26]. The MfpA dimer was selected as the model system for Qnr proteins. Crystal structures were obtained from PDB bank (PDBID: 2BM7) and solvated with TIP3P water molecules [27]. After 1000 steps of energy minimization, the solvent of the system was equilibrated for 200 ps with all protein atoms fixed. Restraints were removed and the system was gradually heated from 25 K to 300 K. The production runs lasted for 10 ns with a time step of 2 fs. Trajectories were saved every 5 ps and data analysis used VMD 1.8.6 [28].

### Homology modeling of QnrC

**The **SWISS-MODEL protein structure homology-modeling server [29] was used to construct the QnrC structure. The residues from 1 to 165, which constitute a tandemly pentapeptide repeat sequence of QnrC, were used in model building. The crystal structure of the typical pentapeptide repeat protein Np275/276 (PDBID: 2J8K) was selected as the template. Model structures were first optimized with 10-ns MD run with all backbone atoms fixed. Then restraints were gradually removed in 5 ns, and the system was further equilibrated for another 20-ns MD simulation.

### Nucleotide sequence accession numbers

The *qnrC *and *qnrA1 *mutant sequences have been submitted to GenBank with accession numbers HM011089 to HM011102 and HM011060 to HM011088.

## List of abbreviations

PRP: pentapeptide repeat protein; MIC: minimal inhibitory concentration; RMSD: root-mean-square deviation; RHQBH: right-handed quadrilateral β-helix; MD: molecular dynamics.

## Authors' contributions

QG performed most biological experiments. JW performed molecular dynamics simulations and homology modeling. XX, MW, XW and XY assisted in experiments. QG and MW conceived and designed the experiments and wrote the manuscript. JW and WW participated in the design of the study and wrote the manuscript. MW and WW supervised the experiments. All authors read and approved the final manuscript.

## Supplementary Material

Additional file 1 **Table S1**: Primers for vector construction and mutagenesis of *qnrC *and *qnrA1 *^a^. Click here for file

## References

[B1] Ling TK Xiong J Yu Y Lee CC Ye H Hawkey PM Multicenter antimicrobial susceptibility survey of Gram-negative bacteria isolated from patients with community-acquired infections in the People's Republic of China Antimicrob. Agents Chemother 2006 50 374 378 10.1128/AAC.50.1.374-378.2006 16377716 PMC1346789

[B2] Martinez-Martinez L Pascual A Jacoby GA Quinolone resistance from a transferable plasmid Lancet 1998 351 797 799 10.1016/S0140-6736(97)07322-4 9519952

[B3] Poirel L Liard A Rodriguez-Martinez JM Nordmann P Vibrionaceae as a possible source of Qnr-like quinolone resistance determinants J Antimicrob Chemother 2005 56 1118 1121 10.1093/jac/dki371 16227349

[B4] Rodríguez-Martínez JM Velasco C Briales A García I Conejo MC Pascual A Qnr-like pentapeptide repeat proteins in Gram-positive bacteria J Antimicrob Chemother 2008 61 1240 1243 10.1093/jac/dkn115 18343805

[B5] Sánchez MB Hernández A Rodríguez-Martínez JM Martínez-Martínez L Martínez JL Predictive analysis of transmissible quinolone resistance indicates *Stenotrophomonas maltophilia *as a potential source of a novel family of Qnr determinants BMC Microbiol 2008 8 148 161 10.1186/1471-2180-8-148 18793450 PMC2556341

[B6] Wang M Guo Q Xu X Wang X Ye X Wu S Hooper DC Wang M New plasmid-mediated quinolone resistance gene, *qnrC*, found in a clinical isolate of *Proteus mirabilis* Antimicrob Agents Chemother 2009 53 1892 1897 10.1128/AAC.01400-08 19258263 PMC2681562

[B7] Tran JH Jacoby GA Mechanism of plasmid-mediated quinolone resistance Proc Natl Acad Sci USA 2002 99 5638 5642 10.1073/pnas.082092899 11943863 PMC122823

[B8] Tran JH Jacoby GA Hooper DC Interaction of the plasmid-encoded quinolone resistance protein Qnr with *Escherichia coli *DNA gyrase Antimicrob Agents Chemother 2005 49 118 125 10.1128/AAC.49.1.118-125.2005 15616284 PMC538914

[B9] Vetting MW Hegde SS Fajardo JE Fiser A Roderick SL Takiff HE Blanchard JS Pentapeptide repeat proteins Biochemistry 2006 45 1 10 10.1021/bi052130w 16388575 PMC2566302

[B10] Bateman A Murzin AG Teichmann SA Structure and distribution of pentapeptide repeats in bacteria Protein Sci 1998 7 1477 1480 10.1002/pro.5560070625 9655353 PMC2144021

[B11] Hegde SS Vetting MW Roderick SL Mitchenall LA Maxwell A Takiff HE Blanchard JS A fluoroquinolone resistance protein from *Mycobacterium tuberculosis *that mimics DNA Science 2005 308 1480 1483 10.1126/science.1110699 15933203

[B12] Vetting MW Subray S Hegde SS Hazleton KZ Blanchard JS Structural characterization of the fusion of two pentapeptide repeat proteins, Np275 and Np276, from *Nostoc Punctiforme*: resurrection of an ancestral protein Protein Sci 2007 16 755 760 10.1110/ps.062637707 17384236 PMC2203339

[B13] Buchko GW Ni S Robinson H Welsh EA Pakrasi HB Kennedy MA Characterization of two potentially universal turn motifs that shape the repeated five-residues fold--crystal structure of a luminal pentapeptide repeat protein from Cyanothece 51142 Protein Sci 2006 15 2579 2595 10.1110/ps.062407506 17075135 PMC2242410

[B14] Buchko GW Robinson H Pakrasi HB Kennedy MA Insights into the structural variation between pentapeptide repeat proteins--crystal structure of Rfr23 from Cyanothece 51142 J Struct Biol 2008 162 184 192 10.1016/j.jsb.2007.11.008 18158251

[B15] Vetting MW Hegde SS Blanchard JS Crystallization of a pentapeptide-repeat protein by reductive cyclic pentylation of free amines with glutaraldehyde Acta Crystallogr D Biol Crystallogr 2009 65 462 469 10.1107/S0907444909008324 19390151 PMC2672816

[B16] Ni S Sheldrick GM Benning MM Kennedy MA The 2Å resolution crystal structure of hetL, a pentapeptide repeat protein involved in regulation of heterocyst differentiation in the cyanobacterium Nostoc sp. Strain PCC 7120 J Struct Biol 2009 165 47 52 10.1016/j.jsb.2008.09.010 18952182

[B17] Willmot CJ Maxwell A A single point mutation in the DNA gyrase A protein greatly reduces binding of fluoroquinolones to the gyrase-DNA complex Antimicrob Agents Chemother 1993 37 126 127 8381633 10.1128/aac.37.1.126 PMC187618

[B18] Jacoby G Cattoir V Hooper D Martínez-Martínez L Nordmann P Pascual A Poirel L Wang M *qnr *gene nomenclature Antimicrob Agents Chemother 2008 52 2297 2299 10.1128/AAC.00147-08 18426894 PMC2443900

[B19] Cattoir V Poirel L Nordmann P In-vitro mutagenesis of *qnrA *and *qnrS *genes and quinolone resistance in *Escherichia coli* Clin Microbiol Infect 2007 13 940 943 10.1111/j.1469-0691.2007.01778.x 17627785

[B20] Rodríguez-Martínez JM Briales A Velasco C Conejo MC Martínez-Martínez L Pascual A Mutational analysis of quinolone resistance in the plasmid-encoded pentapeptide repeat proteins QnrA, QnrB and QnrS J Antimicrob Chemother 2009 63 1128 1134 10.1093/jac/dkp111 19357158

[B21] Morais Cabral JH Jackson AP Smith CV Shikotra N Maxwell A Liddington RC Crystal structure of the breakage-reunion domain of DNA gyrase Nature 1997 388 903 906 10.1038/42294 9278055

[B22] Robicsek A Jacoby GA Hooper DC The worldwide emergence of plasmid-mediated quinolone resistance Lancet Infect Dis 2006 6 629 640 10.1016/S1473-3099(06)70599-0 17008172

[B23] Clinical and Laboratory Standards Institute Performance standards for antimicrobial susceptibility testing; seventeenth informational supplement M100-S17 2007 27 Clinical and Laboratory Standards Institute, Wayne, PA 1

[B24] Phillips JC Braun R Wang W Gumbart J Tajkhorshid E Villa E Chipot C Skeel RD Kale L Schulten K Scalable molecular dynamics with NAMD J Comput Chem 2005 26 1781 1802 10.1002/jcc.20289 16222654 PMC2486339

[B25] Feller SE Zhang YH Pastor RW Brooks BR Constant-Pressure Molecular-Dynamics Simulation - the Langevin Piston Method J Chem Phys 1995 103 4613 4621 10.1063/1.470648

[B26] Martyna GJ Tobias DJ Klein ML Constant-pressure molecular-dynamics algorithms J Chem Phys 1994 101 4177 4189 10.1063/1.467468

[B27] Jorgensen WL Chandrasekhar J Madura JD Impey RW Klein ML Comparison of simple potential functions for simulating liquid water J Chem Phys 1983 79 926 935 10.1063/1.445869

[B28] Humphrey W Dalke A Schulten K VMD - Visual Molecular Dynamics J Molec Graphics 1996 14 33 38 10.1016/0263-7855(96)00018-5 8744570

[B29] Arnold K Bordoli L Kopp J Schwede T The SWISS-MODEL Workspace: A web-based environment for protein structure homology modelling Bioinformatics 2006 22 195 201 10.1093/bioinformatics/bti770 16301204

