# Supplementary material for: A mutational analysis and molecular dynamics simulation of quinolone resistance proteins QnrA1 and QnrC from Proteus mirabilis
Source: BMC Struct Biol. 2010 Oct 8;10:33. doi: 10.1186/1472-6807-10-33 (PMC2964730; doi:10.1186/1472-6807-10-33)
Supplement: Additional file 1 — Table S1: Primers for vector construction and mutagenesis of qnrC and qnrA1 a. [file 1472-6807-10-33-S1.DOC]

Table S1 Primers for vector construction and mutagenesis of *qnrC* and *qnrA1* ^a^

| Primer | | Sequence^c^ | |
| --- | --- | --- | --- |
| qnrA1Sal | | ctagtcgacGccaaatcgaaccttattag | |
| qnrA1Eco | | ctaGAAttcGcagagaaacggcattcc | |
| QnrC-∆11-20-F^b^ | | | CCCATAAAACGTACGATCAA ---CATCACTTTTCTCACTG |
| QnrC-∆11-20-R | | | CAGTGAGAAAAGTGATG---TTGATCGTACGTTTTATGGG |
| QnrC-∆49-55-F | | | CATGGGTTGTACATT---TGGATGTAATTTTATCTATGC |
| QnrC-∆49-55-R | | | GCATAGATAAAATTACATCCA---AATGTACAACCCATG |
| QnrC-∆77-96-F | | | GAATTGCATGCTTTCAATGGC---TAATTTCTCACAGGC |
| QnrC-∆77-96-R | | | GCCTGTGAGAAATTA---GCCATTGAAAGCATGCAATTC |
| QnrC-∆137-156-F | | | AAGCAATGCCTTGAAAAG---TCAGACTTAAGTAGG |
| QnrC-∆137-156-R | | | CCTACTTAAGTCTGA---CTTTTCAAGGCATTGCTT |
| QnrC-∆216-218-F | GGAGCAGTTGGGGGTGATTGTT---AAAGTGTTTTGACCTACG | | |
| QnrC-∆216-218-R | CGTAGGTCAAAACACTTT---AACAATCACCCCCAACTGCTCC | | |
| QnrA1-∆2-10-F | | | GATTAAAGGAAGCCGTATG---gaggatttctcacgcc |
| QnrA1-∆2-10-R | | | ggcgtgagaaatcctc---CATACGGCTTCCTTTAATC |
| QnrA1-∆2-21-F | GATTAAAGGAAGCCGTATG---CGTTTTCGCCGCTGCCGCTTT | | |
| QnrA1-∆2-21-R | AAAGCGGCAGCGGCGAAAACG---CATACGGCTTCCTTTAATC | | |
| QnrA1-∆41-56-F | GCCACTGTCAGCTGCAGGAT---TGTCACTTCAGCTATGCCG | | |
| QnrA1-∆41-56-R | CGGCATAGCTGAAGTGACA---ATCCTGCAGCTGACAGTGGC | | |
| QnrA1-∆51-56-F | | | ggattgcagtttcattgaa---Tgtcacttcagctatgccg |
| QnrA1-∆51-56-R | | | cggcatagctgaagtgacA---ttcaatgaaactgcaatcc |
| QnrA1-∆207-218-F | GCCTGGCAACAGGAGCAA---TAGCTCGAATGCAAACACAAG | | |
| QnrA1-∆207-218-R | CTTGTGTTTGCATTCGAGCTA---TTGCTCCTGTTGCCAGGC | | |
| QnrA1-∆187-218-F | CTTTGCCGATCTGGATGGG---TAGCTCGAATGCAAACACAAG | | |
| QnrA1-∆187-218-R | CTTGTGTTTGCATTCGAGCTA---CCCATCCAGATCGGCAAAG | | |

^a^ Primers for single amino acid mutations are not listed, nucleotide codons for mutant residues are shown in parentheses of Table 1.

^b^ ∆11-20 indicates the deletion mutation of residue 11-20.

^c^ Dashes indicate the deleted nucleotides which encodes the corresponding amino acids of QnrC or QnrA1.
